# Statistics of the Dental Art in Paris in 1828

**Published:** 1841-12

**Authors:** F. Maury, J. B. Savier

**Affiliations:** Paris.


### ARTICLE VI.

*Statistics of the Dental Art in Paris in* 1828. From le traité complete du l'art du dentiste, par F. MAURY, Paris. Translated by J. B. SAVIER.

WE have thought that in the course of our work, it would be interesting to our readers to offer some statistical considerations of the dental art in Paris. This art has really made such a great progress within the last thirty years, that on considering all means employed for the health and cleanliness of the mouth, it produces an annual income (as we shall show) of about six millions of francs.

There were in Paris in 1790, only five dentists; in 1814 there were twenty; and in 1828 there were about one hundred and forty, which we will arrange into ten classes according to their annual receipts.

| Classes. | Number of each Class. | Annual Receipts. | Total of each Class. |
|---|---|---|---|
| 1 | 5 | 40,000 | 200,000 |
| 2 | 6 | 30,000 | 180,000 |
| 3 | 6 | 25,000 | 150,000 |
| 4 | 8 | 20,000 | 160,000 |
| 5 | 8 | 15,000 | 120,000 |
| 6 | 12 | 12,000 | 144,000 |
| 7 | 15 | 9,000 | 135,000 |
| 8 | 20 | 6,000 | 120,000 |
| 9 | 25 | 4,000 | 100,000 |
| 10 | 35 | 2,000 | 70,000 |

Total 140 who receive annually . . . 1,379,000 fr.

We can enumerate in the different districts two hundred dentists who make annually five thousand francs each, making a total of one million of francs.

The apothecaries, perfumers, mercers, and other merchants in

A more perceptible galvanic action is produced by filling different teeth in the same mouth with different metals. We have met with several cases similar to the one mentioned by Dr. Mackall, in a communication published in the first volume of our journal. It is seldom felt however, except in highly irritable dispositions.—*Balt. Ed.*

Paris and other parts of France, by vending powders, elixirs, opiates, and other things employed for the health and beauty of the mouth, receive annually an amount equal to that of the dentists of Paris and the districts, . . . . . . . 2, 379,000 francs.

Now if we admit that out of thirty millions of inhabitants of France, there are five hundred thousand, who, regarding the cleanliness of their teeth, spend annually at least seventy-five centimes for a brush; we will then have another total amount of 1,125,000 francs.

Thus we find that the receipts of the dentists of Paris, are

| | | |
|---|---|---|
| annually . . . . . . . . . | 1,379,000 | francs. |
| that of those of the districts . . . . | 1,000,000 | |
| receipts of the apothecaries, &c. . . | 2,379,000 | |
| do. merchants for the sale of brushes, | 1,125,000 | |
| Total, . . . | 5,883,000 | francs. |

In supposing that out of this amount 630,000 francs are spent for operations that are indispensable, such as the extraction of teeth and other operations performed only by men of the art, and which come properly under the province of the surgeon; the dental art, exclusive of the surgical department, occasions an annual expenditure of nearly 5,253,000 francs. This result may appear surprising to some, but we warrant it to be as correct as these sort of calculations can be made.

